# Cumulative Dietary Risk Assessment of Benzophenone-Type Photoinitiators from Packaged Foodstuffs

**DOI:** 10.3390/foods11020152

**Published:** 2022-01-07

**Authors:** Mei-Lien Chen, Chih-Hsien Chen, Yu-Fang Huang, Hsin-Chang Chen, Jung-Wei Chang

**Affiliations:** 1 Institute of Environmental and Occupational Health Sciences, School of Medicine, Campus of Hsinchu, National Yang Ming Chiao Tung University, Hsinchu 30010, Taiwan; mlchen@ym.edu.tw; 2 Institute of Environmental and Occupational Health Sciences, School of Medicine, Campus of Taipei, National Yang Ming Chiao Tung University, Taipei 11221, Taiwan; 3 Institute of Food Safety and Health Risk Assessment, National Yang Ming Chiao Tung University, Taipei 11221, Taiwan; ak1040831@gmail.com; 4 Department of Safety, Health and Environmental Engineering, National United University, Miaoli 36063, Taiwan; yfh@nuu.edu.tw; 5 Institute of Food Safety and Health, National Taiwan University, Taipei 11221, Taiwan; hsinchang@ntu.edu.tw

**Keywords:** average daily dose, dietary risk, FaPEx, photoinitiators, ultraviolet-cured ink

## Abstract

Photoinitiators used in ultraviolet-cured ink may migrate from food packaging materials into food products. Therefore, we conducted a dietary risk assessment of exposure to benzophenone (BP)-type photoinitiators by quantifying and reducing uncertainties associated with the risk characterization. A total of 362 food packaging samples including 180 cereals, 136 fruit and vegetable juices, and 46 milk samples were subjected to fast pesticides extraction to determine photoinitiator residues. The average daily dose (ADD) of BP was the highest in the age group of zero to three years, with a P97.5 ADD of 2.56 × 10^−4^ mg/kg bw/day. The ADD of 2-hydroxybenzophenone (2-OHBP) was the highest in the age group of three to six years, with a P97.5 UB ADD of 3.52 × 10^−5^ mg/kg bw/day. The estimated UB P97.5 ADD for each age group was below the toxicological concern threshold of 0.0015 mg/kg bw/day. The cumulative toxicity of all BPs, evaluated using the MOE_T_ value, was at an acceptable level. Although the MOE_T_ value of BPs was above the safety limit in the foodstuffs studied herein, this result may be different if Taiwan were to follow regulation guidelines for BP-type photoinitiators based on the specific migration limit for the unmeasured BP residues in other foodstuffs.

## 1. Introduction

Benzophenone (BP)-type ultraviolet (UV) filters are widely used in inks and in personal care products such as perfumes and soaps to reduce damage to the odors and color of the products from UV irradiation. Furthermore, because food packaging materials are often exposed to solar UV light, BP may be added to polymers used in food packaging materials to prevent degradation of the packaging, the food product inside, or both. Such packaging materials can be transparent and protect both the food and the packaging [1,2].

Despite the advantages of packaging materials with BP, such packaging is a potential source of chemical contamination, thus leading to debates concerning its environmental and health effects. BP is not completely stabilized during the printing process or removed after the process, as a result of which, it is not firmly bound to the print film layer [3]. When the carton board that carries these packaged foodstuffs are pressed and crushed, BP may migrate into food from the packaging material, either in the form of a powder or in the gaseous phase, thus exposing the food products to BP [4,5]. The migration of such compounds to foods may endanger the health of consumers or cause unacceptable changes in the composition or organoleptic characteristics of food [6].

In 2009, the EU Rapid Alert System for Food and Feed reported a photoinitiator pollution episode characterized by unusually high levels of 4-methylbenzophenone (4-MBP) in some breakfast cereal products (chocolate crunch) [7]. Repeated analysis by the manufacturer confirmed high levels of 4-MBP in these products (>4200 μg/kg BP) [8].

The EU Commission stated that printing inks mixed with 4-MBP and BP are unsuitable for use in printing on food packaging unless a protective interface is incorporated in the packaging that prevents the transformation of these compounds into the gas phase and blocks their migration into food. The committee established a cumulative limit of 0.6 mg/kg for BP and 4-MBP [7]. According to EU Directive 2002/72/EC, BP can be used as an additive in plastic materials, with a specific migration limit (SML) of 0.6 mg/kg [9].

Several BP-type photoinitiators have been detected in breakfast cereals, milk, and packaged fruit juices, including BP, 2-hydroxybenzophenone (2-OHBP), 4-hydroxybenzophenone (4-OHBP), 4-MBP, methyl-2-benzoylbenzoate (M2BB), and 4-phenylbenzophenone (PBZ). Toxicological studies have evidenced that the photoinitiator BP is carcinogenic and causes reproductive toxicity in animals. BP is listed as a group 2B carcinogen (possibly carcinogenic) by the International Agency for Research on Cancer. Several countries have set SMLs for photoinitiator substances on food packaging contact materials and inks [10,11,12]. However, Taiwan currently has no regulation for the use of BP substances in food contact materials and inks, and no report has assessed the health risk of exposure to BP from typical foodstuffs in Taiwan. Therefore, in this study, the RISK21 matrix was used to evaluate the noncarcinogenic and carcinogenic risks of BPs ingested through breakfast cereals, fresh milk, and packaged fruit juices in different age groups.

The RISK21 matrix is a simple, efficient, and transparent integrated risk assessment strategy that emphasizes a systematic tier risk assessment process structure based on problem formulation and driven by exposure assessment. The RISK21 matrix was first applied to the existing data for exposure and toxicological assessments, and then the exposure concentration and toxic effects were compared in a visualization matrix to assess the degree of health concern of the hazard. The RISK21 matrix can concretely and visually present the risk assessment results, which may help in subsequent decision-making and communication regarding the risks and provide a breakthrough in risk assessment criteria and methodology [13,14].

The Threshold of Toxicological Concern (TTC) approach is used in the early assessment of the safety of chemicals that lack complete toxicological data. The TTC provides conservative exposure limits based on the chemical structure and toxicological information of related chemicals [15]. The TTC approach is widely applied in the risk assessment of chemicals in different materials, including food contact materials (FCMs). The feasibility of the TTC approach in the risk assessment of chemicals in FCMs has been established by both the US FDA and European Food Safety Authority (EFSA) [16,17]. Hence, this approach was adopted in this study as an early warning tool to detect BPs that lacked complete toxicological data, thus enabling prompt remedial action.

Fast pesticide extraction (FaPEx) is a method used to extract pesticide residues in agricultural samples by using single-use prefilled sealed cartridges; this method is innovative, simple, and fast and is a simplified version of the QuEChERS method that is based on the same principles [18]. We have developed a FaPEx technique coupled with ultra-HPLC (UHPLC)-MS/MS to simultaneously analyze the levels of targeted BPs in the present study.

## 2. Materials and Methods

### 2.1. Chemicals and Reagents

Acetonitrile (ACN) and methanol for high-performance liquid chromatography (HPLC) were obtained from Merck (Darmstadt, Germany). Analytical standards, namely 4-OHBP (1137-42-4), M2BB (606-28-0), BP (119-61-9), 2-OHBP (117-99-7), 4-MBP (134-84-9), and PBZ (2128-93-0), were obtained from Sigma-Aldrich (St. Louis, MO, USA), as were formic acid (≥95%) and HPLC-grade methanol (≥95%). Internal standards (i.e., d_4_-4-OHBP, di-2-OHBP, d_5_-BP, and d_3_-4-MBP) were obtained from Cambridge Isotope Laboratories (Andover, MA, USA). Milli-Q water was purified using a Millipore system (Billerica, MA, USA).

### 2.2. Food Sampling

The selected criteria of foodstuffs was based on Taiwanese dietary habits derived from the Nutrition and Health Survey in Taiwan (NAHSIT) [19]. The primary aim of NAHSIT is to establish a long-term, stable and regular monitoring system, which can monitor the national health and food consumption for all ages, and then face-to-face interviews were conducted. In the NAHSIT, a multistage, stratified, probability sampling design was employed to select participants representative of the Taiwanese population.

The first criterion was the four-level food classification of cereals in the NAHSIT, and the second was classification according to grain type, namely whole grain rice flour (cereal I), whole grain wheat and its products (cereal II), and whole grain processed grain products (cereal III). A total of 362 food packaging samples including 180 cereal samples, 136 fruit and vegetable (F&V) juice samples, and 46 milk samples, packaged in plastic material and purchased from a supermarket and traditional market.

Otherwise, the BP levels were also evaluated in cereal samples with different packaging, such as iron and aluminum cans, aluminum foil, plastic, and paper boxes. We preferentially selected iron and aluminum cans as the grain substrate and paper packaging materials as tested food samples. In addition, for each packaging material, the sales ranking of the relevant brand at physical stores and on online platforms was reviewed. Overall, we collected 59 samples of cereal I, 61 samples of cereal II, and 60 samples of cereal III.

We selected 182 F&V juice and milk samples according to the food classification of the NAHSIT. For more comprehensive results, the F&V juice samples were categorized into three groups: (i) 100% fresh F&V juice, (ii) 100% reconstituted F&V juice, and (iii) 10% or more F&V juice. The milk samples were classified as (i) full-fat milk and (ii) low-fat milk. On the basis of the material used, the packaging of the F&V juices was categorized as refrigerated carton board packaging, aseptic carton packaging (aluminum foil), and plastic bottles. Milk packaging primarily comprised refrigerated carton board and plastic bottles, with a few packaging materials being glass bottles and aluminum-tin cans. One study indicated that using aluminum-tin cans and aseptic cartons as packaging materials can effectively inhibit the migration of BP into F&V juices because such materials are multilayered and contain aluminum foil [4]. Therefore, F&V juice and milk samples that were mainly stored in refrigerated cartons and plastic bottles were collected. In addition, we collected F&V juices and milk stored in glass jars to serve as controls, because BP can migrate through aluminum-tin cans and aseptic carton packaging into F&V juices, thus rendering those materials unsuitable to be considered as control. In total, we collected 136 F&V juice samples (including 14 100% fresh F&V juice samples, 63 100% reconstituted F&V juice samples, and 59 10% or more F&V juice samples) and 46 milk samples (including 37 full-fat and nine low-fat milk samples)

### 2.3. Sample Pretreatment

To extract BPs from foods, the fast pesticide extraction (FaPEx) method, a novel and rapid technique for extraction of pesticide residues in agricultural products, was used [18]. FaPEx is currently the fastest method available worldwide. This technique was developed by the Taiwan Agricultural Chemicals and Toxic Substances Research Institute and was granted a patent by the Taiwan government in 2015. FaPEx is easier to apply and more efficient than the QuEChERS method, which is meant as the quick, easy, cheap, effective, rugged, and safe method. FaPEx has the following benefits: three-fold higher total efficiency than comparable methods, use of less operating equipment, 60% reduction in labor costs, use of at least 50% less organic solvents, and low wastage. The following process was followed for sample extraction: (i) 0.5 g of finely crushed cereal was added to a 15-mL centrifuge tube, followed by the addition of 1 mL of water and 20 μg/L of the BP IS; the mixture was then allowed to stand for 30 min. (ii) 5 mL of ACN was mixed with 1% acetic acid and vortexed for 5 min, and the mixture was then centrifuged for 10 min at 5500 rcf. (iii) Subsequently, 5.5 mL of the supernatant was passed through a single-use FaPEx-CER cartridge, and the extract was allowed to flow via gravity. (iv) The supernatant was filtered, and 1 mL of the sample extract was transferred to a vial. (v) Finally, ultrahigh-performance liquid chromatography tandem mass spectrometry (UHPLC-MS/MS) was performed. The details of the method have been described in our earlier study [20] and supplemental data.

### 2.4. Instrumental Analysis

Chromatographic analysis was performed using a Waters ACQUITY UPLC BEH C18 column (2.1 mm × 100 mm, 1.7 μm), and quantification was then conducted through UHPLC-MS/MS (LCMS 8045, Shimadzu). The flow rate was set to 0.3 mL/min. The gradient eluent consisted of mobile phase A (ultrapure water) and mobile phase B (methanol mixed with 0.1% formic acid). The injection volume was 10 μL. The optimal chromatographic gradient program was as follows: from 20% to 80% B over 0–3.5 min, maintenance at 80% B for 3.5–4.5 min, from 80% to 90% B over 4.5–5.5 min, maintenance at 90% B for 5.5–9.5 min, from 90% to 20% B over 9.5–9.6 min, and maintenance at 20% B to 13.5 min. The following six printing inks and photoinitiators were analyzed through UHPLC-MS/MS: BP, 2-OHBP, 4-OHBP, 4-MBP, M2BB, and PBZ. The performance of UHPLC-MS/MS was monitored on the basis of the recovery of the surrogate standards in each of the samples. To monitor the method efficiency of each batch, 30% of the analyzed samples were used for quality control as matrix-spiked samples fortified with a known amount of the target analyte; 10% of the analyzed samples were used as random duplicate samples.

Each pool sample that was not spiked was analyzed in duplicate. In addition, in each batch of samples, a procedural blank was included to control for background contamination. The analytes in pooled samples were quantified using the calibration curve obtained through the standard addition method by plotting the peak area/internal standard ratio versus the added amount of each standard. The use of this method prevented the matrix effect (ME) during the quantitation.

### 2.5. Validation Procedure

The proposed method was validated according to guidelines established in the United States [21] and Taiwan [22] on the basis of evaluations of linearity, the ME, the limit of detection (LOD), the limit of quantification (LOQ), precision, and accuracy. We described these detailed QA/QC procedures in a previous study [19]. In addition, the retention time, MS parameters (e.g., ion transitions for quantification and confirmation), and collision energy of BPs obtained in the MRM mode and the chromatographic conditions were optimized [20].

### 2.6. ME and Process Efficiency

Initially, all procedures were evaluated in terms of the ME through a comparison of the areas of the standard between the extract and the solvent, as follows: ME (%) = (area of the standard in the matrix/area of the standard in the solvent) × 100. An ME of approximately 100% indicated no effect of the matrix, whereas an ME of 80–120% indicated a substantial ME. For the validated method, which did not involve cleanup, the ME was calculated as follows: ME (%) = ([slope in the matrix/slope in the solvent] − 1) × 100. Negative values of MEs indicated the suppression of the signal, and positive values indicated enhancement. Values <20% indicated no ME or low ME, and values >20% indicated a high ME [23].

### 2.7. Hazard Characterization and Health-Based Guidance Values

A health-based guidance value (HBGV) has been established for only one of the detected photoinitiators, namely BP. Its TDI (Tolerable daily intake) was set as 0.03 mg/kg bw/day (30 μg/kg bw/day) by the EFSA in 2009 according to the nonneoplastic kidney effects in male rats in a chronic carcinogenicity study with a UF of 100 for interspecies and intraspecies differences [24]. Regarding the other photoinitiators, in 2009, the European Chemicals Agency (ECHA) and EFSA conducted a risk assessment of three photoinitiators (4OHBP, M2BB and PBZ) and one photoinitiator (4MBP) for animals, respectively. However, evidence from animal studies for 2-OHBP is lacking; hence, the TTC approach was used to characterize dietary risk. The Cramer decision tree can be used to estimate the toxicological hazard of a compound according to its molecular structure. For this purpose, Toxtree v3.1.0 (Ideaconsult Ltd., Sofia, Bulgaria) software was used [25]. First, on the basis of the Benigni/Bossa rule base (for mutagenicity and carcinogenicity), 2-OHBP was found to be negative for genotoxic and nongenotoxic carcinogenicity. Thereafter, the method was used to classify molecules into three classes: class I (low toxicity), class II (intermediate toxicity), and class III (high toxicity). The thresholds for Cramer classes I–III were 1800, 540, and 90 μg/person/day, respectively [26]. A summary of this approach and its application to printing inks/photoinitiators is presented in Table S1. The dietary burden and classification of 2-OHBP detected according to the TTC approach are presented in Table S2.

The maximum concentration of compounds detected that was considered sufficiently conservative was retained in the assessment to ensure that outcomes remained protective of human health. The EFSA’s Panel on Food Contact Materials, Enzymes and Processing Aids considered the effects of nonneoplastic kidney diseases, as observed in a chronic assay. A benchmark dose (BMD) analysis was performed to examine the effects of nonneoplastic kidney disease on male rats, and the lower 95% confidence limit of the BMD for a 10% effect (BMDL_10_) was calculated to be 3.1–7.4 mg/kg bw/day. Through the application of an uncertainty factor of 100, a TDI of 0.03 mg/kg bw was obtained by the panel. The panel had also established a group TDI of 0.01 mg/kg bw for BP and 4-OHBP. However, according to the EFSA, in the absence of supporting data, this fact alone does not justify the inclusion of 4-OHBP in the same group as that of BP based on TDI.

The Panel on Food Contact Materials, Enzymes and Processing Aids used BMD analysis to examine the effects of neoplastic kidney disease resulting from exposure to BP on male rats. The BMDL_10_ was 18.5 mg/kg bw/day. Several studies have indicated that BP is a nongenotoxic carcinogen; thus, at the aforementioned mean value, BP is a threshold carcinogen [27,28].

The nonobserved adverse effect level (NOAEL) of 100 mg/kg bw/day of 4-OHBP was determined using the 28-day repeated-dose toxicity test [29]. According to the Panel on Food Contact Materials, Enzymes and Processing Aids, the toxicity data on 4-MBP are incomplete. A BMDL_10_ of 3.1 mg/kg bw/day for BP was used; however, two additional uncertainty factors were considered [24]. We examined the implantation site effect of PBZ at a dose of 1000 mg/kg bw/day of PBZ in rats by performing the 28-day repeated-dose toxicity test. The NOAEL was 300 mg/kg bw/day at this endpoint [30]. On the basis of the results of the 28-day repeated-dose toxicity test, an NOAEL of 31.25 mg/kg bw/day of M2BB for kidney degeneration and hyperplasia was established by ECHA [31]. All toxicological data of the six BPs are listed in Table S1.

### 2.8. Exposure Assessment

#### 2.8.1. Exposure Scenario

**Figure 1:**
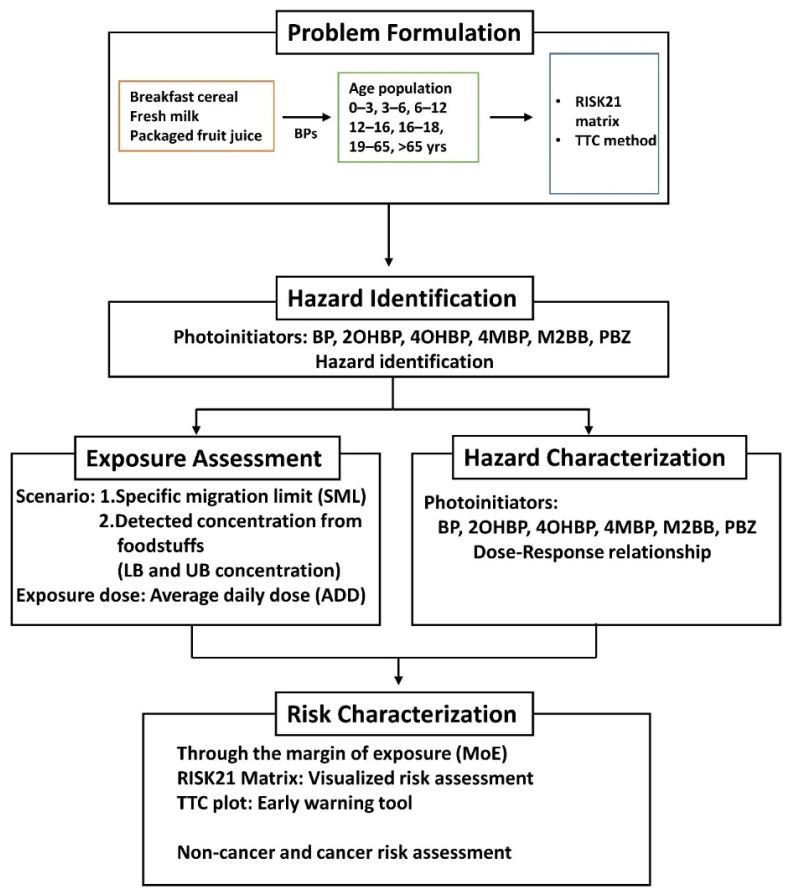
Steps of the risk assessment performed in this study. BP = benzophenone; 2-OHBP = 2-hydroxybenzophenone; 4-OHBP = 4-hydroxybenzophenone; 4-MBP = 4-methylbenzophenone; M2BB = methyl-2-benzoylbenzoate; PBZ = 4-phenylbenzophenone; TTC = threshold of toxicological concern; LB = lower bound; UB = upper bound.

Two exposure scenarios were constructed to calculate the exposure dose in this study (Figure 1). In scenario 1, the SMLs of four BP photoinitiators (BP: 0.6 mg/kg, 4MBP: 0.05 mg/kg, M2BB: 0.05 mg/kg, PBZ: 0.01 mg/kg), derived from the Swiss Ordinance SR817.023.21, were used. Because no SML data are available for 2-OHBP and 4OHBP, these compounds were not included in the calculation of exposure dose and risk in scenario 1. In scenario 2, the measured concentrations of BP and BP derivatives in breakfast cereal, fresh milk, and packaged F&V juice were used. The food sampling details are described in Section 2.2. The analyte data were examined using the lower bound (LB) and upper bound (UB) approach based on the EFSA’s recommendation [32]. At the LB, the concentration values below the LOQ and the LOD were equal to zero. At the UB, the concentration values below the LOD/LOQ were equal to the LOD/LOQ values.

To calculate the dietary intake of BPs, daily consumption amounts were first multiplied by the UB and LB concentrations for each food type. Because the detection rate of BP was 100% in this study, BP was not quantified using the LB and UB approach in scenario 2. To further calculate daily intake (in mg/kg bw/day), the average weights of the people of each sex and in each age group were used; values were also obtained from the Nutrition and Health Survey in Taiwan (NAHSIT), a nationwide representative survey that investigated and monitored the nutritional status of Taiwanese people. The survey method has been described elsewhere [19].

#### 2.8.2. Exposure Assessment

The exposure assessment procedure is described in Figure 1. The average daily dose (ADD) of BP was estimated on the basis of the intake rate of foods from an age-specific NFCD derived from the NAHSIT between 2013 and 2016 and by using the measured concentration of BP in corresponding food items. The equation is as follows:

(1) $$\mathrm{ADD}=\frac{C\;\times \mathrm{IR}\;\times \mathrm{AF}}{\mathrm{BW}}\times 10^{-6}$$

where ADD (mg/kg bw/day) is the average daily dose. C (ng/g or ng/mL) differs in the two exposure scenarios. In scenario 1, we used the SMLs of four BP-type photoinitiators from the Swiss Ordinance SR817.023.21 (BP: 0.6 mg/kg, 4MBP: 0.05 mg/kg, M2BB: 0.05 mg/kg, and PBZ: 0.01 mg/kg). In scenario 2, we used the concentration of the six BP-type photoinitiators in cereal, fresh milk, and packaged F&V juice samples obtained using the LB and UB approach. Ingestion rate (IR; g/day) is the intake rate of food per day (Table S3), and AF (%) is the absorption factor of BP-type photoinitiators. In this study, we conservatively assumed AF to be 100% for each BP-type photoinitiator. BW (kg) is body weight. The conversion factor of units is 10^−6^.

We calculated ADD by using a probabilistic approach. Intake was calculated using ^@^RISK software (from Palisade Coporation, Ithaca, NY, USA), a Monte Carlo computational system for stochastic modeling of dietary exposure. We determined the exposure of a randomly selected person in the consumption database by multiplying the quantity of each relevant food product consumed by the individual in one day by a randomly selected concentration per commodity from the concentration database. According to the raw data on BP concentrations in foods, we used the Kolmogorov–Smirnov test to predict the best-fitting distribution (i.e., normal and lognormal) of the parameters. Considering the lack of raw data of IR and BW parameters, we set a lognormal distribution and normal distribution, respectively [33]. A Monte Carlo simulation was performed with 10,000 iterations to ensure stability. Different possible outcomes generated iteratively were assembled to create a probabilistic statement of the range of results obtained. To conduct a more conservative risk assessment, we selected ADDs of P50 and a P97.5 for the risk characterization. The daily intake distribution was thus generated, including variability and uncertainties. In addition, we conducted a sensitivity analysis using Monte Carlo simulation to determine how various sources or input values of an individual variable affect a specific dependent variable under an allotted group of assumptions.

### 2.9. Risk Characterization

The results of the hazard characterization and exposure assessment were integrated as aforementioned, the critical endpoint and point of departure (POD) of each BPs were selected, and P50 and P97.5 ADDs were derived from the SML (scenario 1) and UB concentration (scenario 2) for use in the risk characterization. In this study, the RISK21 matrix was used for risk characterization. The RISK21 matrix is a tool for determining the exposure and toxicity doses. The risk value was calculated using the MOE. The MOE is a quantitative tool used to determine potential risk arising from exposure to a pesticide active ingredient. An MOE is defined as the ratio of the critical POD to estimated human exposure. The resulting value is compared with the acceptable or target MOE. Values at or above the target MOE are generally considered protective against toxicity. The general target MOE of 100 is considered appropriate for interspecies and intraspecies uncertainty factors.

Finally, the same endpoint of BP-type photoinitiators was selected to derive the combined MOE (MOE_T_), including that for BP, 4-MBP, and M2BB. The MOE_T_ is the reciprocal of the sum of the reciprocals of the MOEs of each chemical being combined [34]. The equation is as follows:

(2) $${\mathrm{MOE}}_{T}=\frac{1}{\frac{1}{{\mathrm{MOE}}_{\mathrm{BP}}}+\frac{1}{{\mathrm{MOE}}_{4\mathrm{MBP}}}+\frac{1}{{\mathrm{MOE}}_{M2\mathrm{BB}}}}$$

## 3. Results

### 3.1. Method Validation

The recoveries of the six spiked samples are listed in Table S4. For the cereal samples, the ME met the criteria, with the exception of that for 2-OHBP in cereal I, and the ME for PBZ in cereal II did not fall within the range of 80–120%. For F&V juices, the ME was 82–110%. For fresh milk, the ME was 99–103%. Therefore, almost all analytes exhibited recoveries between 80% and 120%. For the cereal samples, the recoveries of the three classes of grains were primarily between 80% and 120%. However, the recoveries for M2BB, 4-MBP, and PBZ in cereal I; M2BB in cereal II; and M2BB and PBZ in cereal III were not between 80% and 120%; the addition of IS quantification could compensate for this result. For the F&V juice samples, the recoveries of the six BPs were 95–106%. Moreover, for fresh milk, the recovery rate (accuracy) of the six BPs was 96–110%. All analytes had an RSD value of <20%, indicating high accuracy of the quantification. The RSD values of the cereal samples were <20%, except those for BP and 2-OHBP in the cereal II and III groups on different days. The RSD values of F&V juice samples and milk samples were within the ranges of 6.60–10.3% and 3.60–11.8%, respectively. Chromatographic responses at signal-to-noise ratios of three and 10 were used to determine the LOD and LOQ, respectively. The minimum LOD for BP analytes was <0.001 ng/g in cereal samples and <0.033 and <0.065 ng/mL in F&V juice and milk samples, respectively. According to the guidelines of the Taiwan Food and Drug Administration for the validation and verification of quantitative and qualitative testing methods, the method demonstrated high accuracy and precision, and the ME was 80%–120%; however, the ME was not significant. These results indicated that the method used in the study could be beneficial for assessing breakfast cereals, F&V juices, and milk.

### 3.2. BP Levels in Breakfast Cereals with Different Packaging Materials

Among the three types of cereals, cereal III samples generally had the highest mean BP levels (89.1 ng/g), followed by cereal I (37.6 ng/g) and II (21.0 ng/g) samples (Table 1). The highest 4-MBP levels were generally found in cereal II samples. In addition, BP and 4-MBP were detected in 17 grain samples packaged in iron and aluminum cans, with detection rates of 100% and 88%, respectively (Table S5). BP, 4-MBP, 4-OHBP, and PBZ were detected in 108 grain samples packaged in aluminum foil, with detection rates of 100%, 94%, 8%, and 1%, respectively. Furthermore, BP, 4-MBP, 4-OHBP, M2BB, and PBZ were detected in 55 grain samples packaged in plastic, with detection rates of 100%, 98%, 7%, 9%, and 2%, respectively.

In cereals, the arithmetic mean (AM) concentrations (range) of BP were 26.9 (14.4–71.4), 29.6 (13.6–109), and 95.0 (14.2–1084) ng/g, respectively, in iron–aluminum can, aluminum foil, and plastic packaging materials. In addition, the AM concentrations (range) of 4-MBP were 3.83 (1.21–10.0), 2.33 (1.03–1.9), and 4.13 (0.90–65.8) ng/g, respectively, in iron–aluminum can, aluminum foil, and plastic packaging materials.

The BP concentration in plastic packaging materials was significantly higher than that in iron–aluminum can and aluminum foil packaging materials. After the exclusion of extreme values of BP concentration in plastic packaging materials, the geometric mean (GM) concentration in breakfast cereals with plastic packaging was calculated as 40.8 ng/g, which was still higher than that in breakfast cereals with the other two types of packaging materials.

### 3.3. BP Levels in F&V Juices and Milk with Different Packaging Materials

Among the three types of F&V juices, the highest BP, 4-MBP, and 4-OHBP concentrations were generally found in the orange juice samples (Table 1). Orange juice is rich in pulp and fiber, and these analytes, particularly BP and 4-MBP, are easily adsorbed in pulp fibers. Plastic bottle or carton packaging could lead to an increase in the BP levels in F&V juices. The BP concentrations in all the three types of F&V juices ranged from 5.21 to 51.4 ng/mL.

The average BP concentration in milk samples stored in plastic bottles, cartons, Tetra Paks, and glass bottles was 8.10, 8.21, 7.05, and 7.17 ng/mL, respectively. Furthermore, we compared the BP concentration in milk samples with different fat contents (full fat and low fat); the average BP concentration was 8.07 ng/mL in full-fat milk and 8.18 ng/mL in low-fat milk. In addition, the highest concentration of 4-MBP was detected in the 46 milk samples, and most of these samples were packaged in cartons. Surprisingly, the 4-MBP concentration in low-fat milk packaged in cartons was 150 ng/mL. The 4-MBP concentration in low-fat milk packaged in plastic bottles was under the detection limit.

The UB average M2BB concentration was 0.53 ng/mL in 100% fresh F&V juices, 0.48 ng/mL in 100% reconstituted F&V juices, 0.54 ng/mL in 10% or more F&V juice drinks, 0.17 ng/mL in whole milk, and 0.14 ng/mL in low-fat milk. The M2BB concentration between the F&V juice and milk samples slightly differed; this difference may be attributable to the M2BB concentration present in the food prior to packaging.

### 3.4. ADD of BPs for Different Age Groups

**Figure 2:**
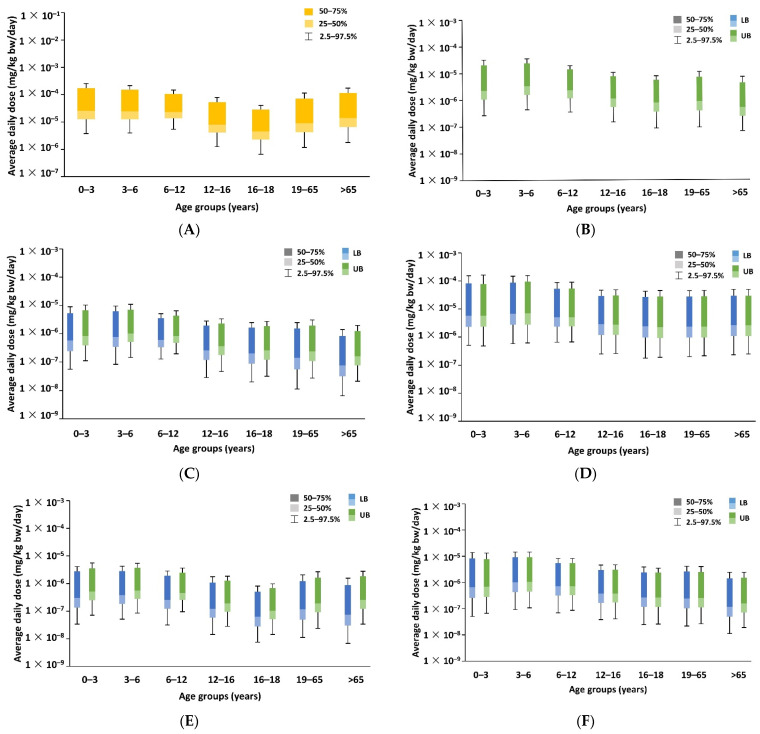
ADD of exposure to BP-type photoinitiators in different age groups (scenario 2). (**A**) BP, (**B**) 2-OHBP, (**C**) 4-OHBP, (**D**) 4-MBP, (**E**) M2BB, (**F**) BPZ. LB = lower bound; UB = upper bound.

Dietary exposure of consumers to chemicals is a crucial element in risk assessment. Estimated dietary exposure values (P50 and 97.5 percentile) of selected individuals of the Taiwan population in different age groups are presented in Figure 2. Two scenarios were explored in this study. In scenario 1, the SML of each BP-type photoinitiator, derived from the Swiss Ordinance SR817.023.21, was used, and the corresponding intake rate and body weight were obtained from the NAHSIT in 2019 (Tables S3 and S6). In scenario 2, the concentration of photoinitiators detected in foods and the corresponding intake rate and body weight data from the NAHSIT in 2019 were used to estimate the ADD in the general population for determining exposure to BP-type photoinitiators through the consumption of food (Table 1 and Table S3).

In scenario 1, the ADD of BP exposure in the age groups of zero to three and three to six years was higher than that of the other three BPs, and the P97.5 ADD in both the age groups was 1.12 × 10^−2^ mg/kg bw/day. The ADDs of all BPs in the age groups of zero to three and three to six years were higher than those in other age groups (Table S7). In scenario 2, the ADD of BP, 4MBP, and M2BP were the highest in the age group of 0–3 years, and the P97.5 ADD of BP, 4MBP, and M2BP were 2.56 × 10^−4^, 1.62 × 10^−4^, and 5.55 × 10^−4^ mg/kg bw/day, respectively (Figure 2). The ADDs of 2-OHBP, 4-OHBP, and PBZ were the highest in the age group of 3–6 years, and the P97.5 UB ADDs of 2-OHBP, 4-OHBP, and PBZ were 3.52 × 10^−5^, 1.09 × 10^−5^, and 1.46 × 10^−5^ mg/kg bw/day, respectively.

### 3.5. Sensitivity Analysis for the Age Group of 19–65 Years

Sensitivity analysis was performed to evaluate which input parameters exerted a more dominant effect on the uncertainty in the model output, and the contribution of each input factor to the variance in the output parameter of interest was quantified for the age group of 19–65 years (Figure S1). Overall, the absolute number of people in this age group was the highest in this age group, with members of this group accounting for the largest proportion of the total population. Figure S1 presents the four parameters that exerted the most dominant effects on each BP-type photoinitiator. For BP, body weight had the most dominant effect on the ADD (contribution to variance, 26.8%), followed by the IR of products from the cereal II group (25.20%), IR of 100% reconstituted F&V juices (24.1%), and IR of full-fat milk (13.0%).

For 2-OHBP, the variable that exerted the greatest effect was the IR of full-fat milk (42.8%), followed by body weight (25.4%), the IR of 100% reconstituted F&V juices (17.0%), and the IR of low-fat milk (10.4%).

For 4-OHBP, the variable that exerted the greatest effect was the IR of full-fat milk (47.3%), followed by body weight (22.5%), the IR of low-fat milk (21.5%), and the IR of 100% reconstituted F&V juices (5.8%). For 4-MBP, the variable that exerted the greatest effect was the IR of low-fat milk (35.3%), followed by body weight (18.9%), the IR of full-fat milk (17.9%), and the IR of 100% reconstituted F&V juices (11.9%).

For M2BB, the variable that exerted the greatest effect was the IR of 100% reconstituted F&V juices (37.4%), followed by body weight (25.4%), the IR of products in the cereal II group (14.4%), and the IR of full-fat milk (12.0%). For PBZ, the variable that exerted the greatest effect was the IR of full-fat milk (42.3%), followed by the IR of low-fat milk (19.7%), body weight (19.4%), and the detected concentration of PBZ in full-fat milk (7.7%).

### 3.6. Dietary Risk Characterization

**Figure 3:**
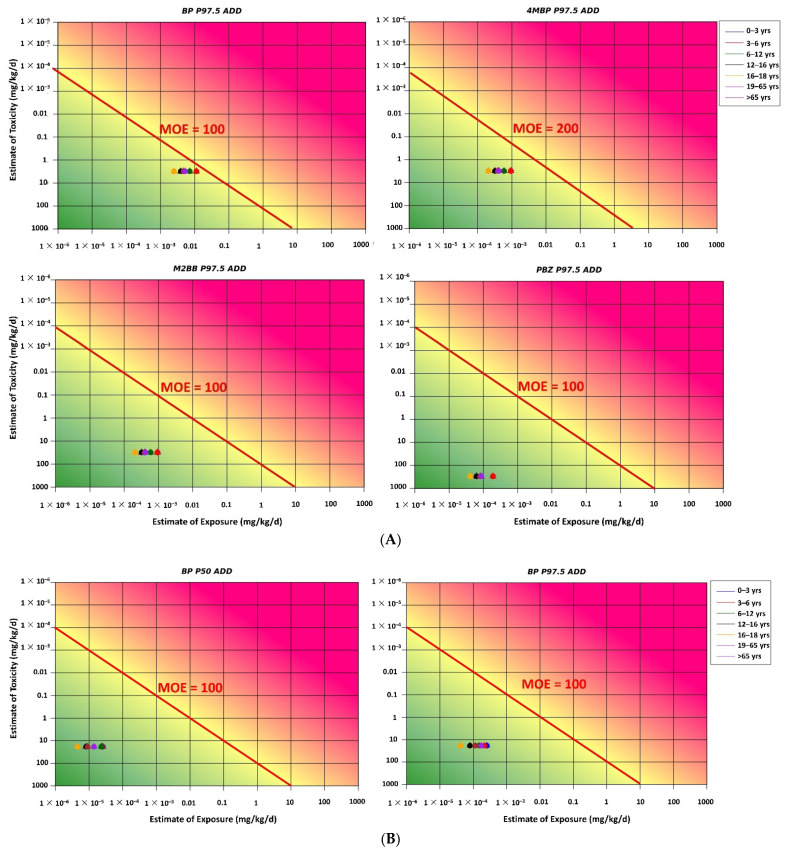
(**A**) Noncarcinogen risk in different age groups determined using the RISK21 matrix (scenario 1). (**B**) Carcinogen risk in different age groups determined using the RISK21 matrix (scenario 1). MOE = margin of exposure; ADD = average daily dose.

**Figure 4:**
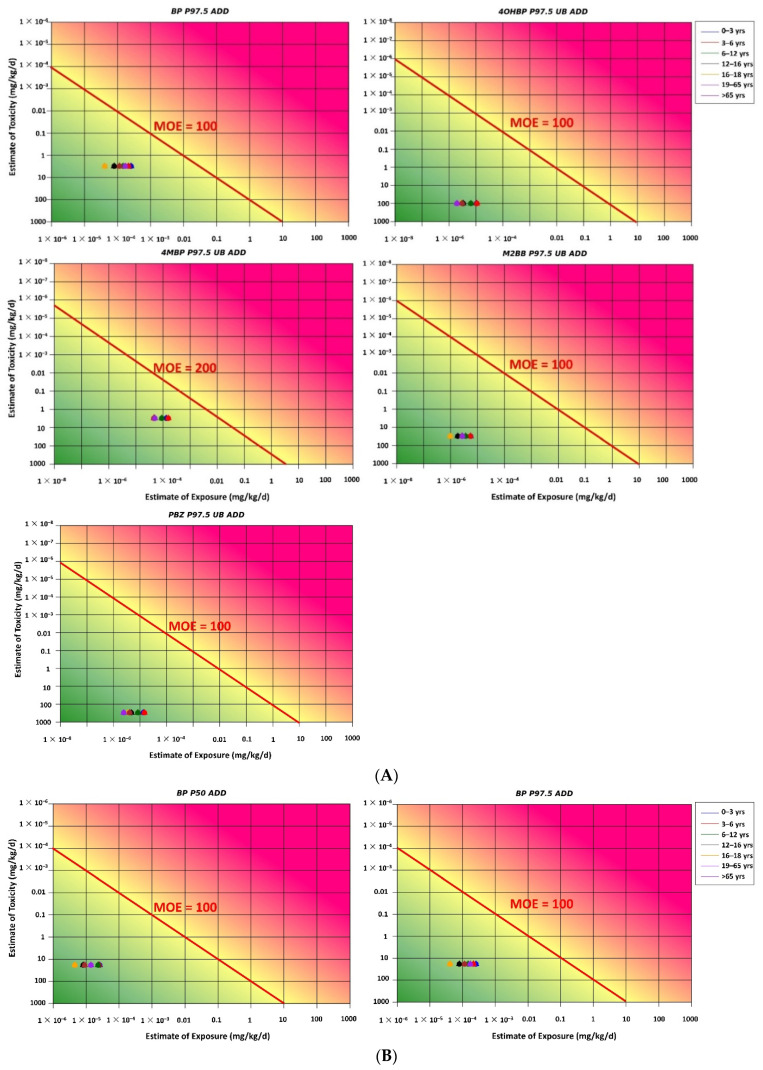
(**A**) Noncarcinogen risk in different age groups determined using the RISK21 matrix (scenario 2). (**B**) Carcinogen risk in different age groups determined using the RISK21 matrix (scenario 2).

**Figure 5:**
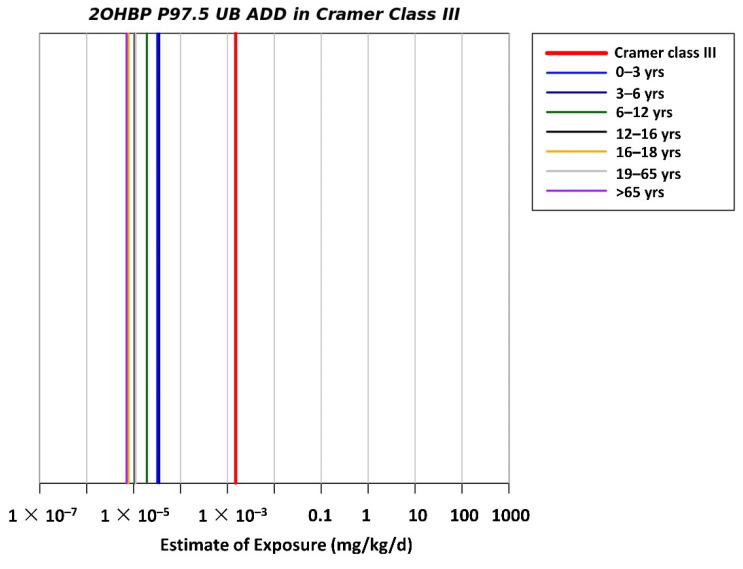
TTC plot of exposure to 2-OHBP in different age groups.

The RISK21 matrix was applied for carcinogen and noncarcinogen risk assessments based on both scenarios 1 and 2. For both the scenarios, all MOE values for each BP-type photoinitiator were above the safety limit, indicating that noncarcinogen and carcinogen risks for humans were within the acceptable range (Figure 3A,B and Figure 4A,B). Because of the lacking toxicological data for 2-OHBP, the TTC approach was used as an early warning tool for risk assessment. The estimated UB P97.5 ADD for each age group was below the TTC value of 0.0015 mg/kg bw/day (Cramer class III), indicating that no additional assessment was required to examine the adverse effect of 2-OHBP on humans (Figure 5).

**Figure 6:**
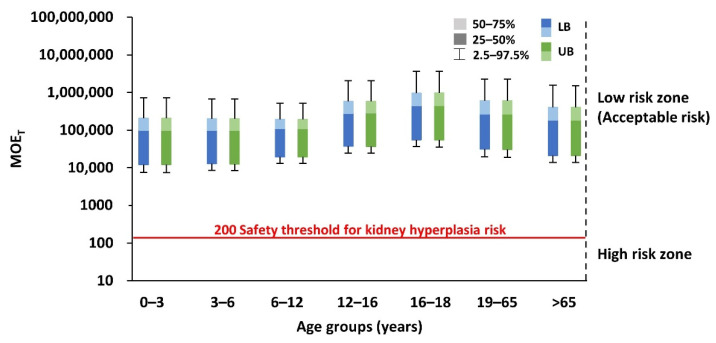
Combined MOE (MOE_T_) of BP, 4-MBP, and PBZ in different age groups.

The estimated MOE_T_ value in all age groups was higher than the target MOE_T_ value of 200 specified by the EFSA for 4-MBP (Figure 6). The result of the cumulative risk assessment indicated an acceptable risk of kidney hyperplasia through exposure to BP, 4-MBP, and M2BB. An assessment of the estimated dietary exposure of BPs compared with the relevant HBGVs indicated no health concerns in any of the age groups studied. Thus, the lifetime cancer risk due to BP exposure was likely to be below 10^−6^ (or one in a million), indicating no significant dietary risk. 

The estimated dietary exposure assessment for 2-OHBP was also based on the TTC class-III threshold, which is 1.5 μg/kg bw/day, and the findings indicated no safety concerns and negligible health risks.

## 4. Discussion

### 4.1. BP Levels in Different Foods and Types of Packaging Materials

In this study, the BP levels of 362 foods from 8 categories were evaluated. The highest BP levels were observed in whole grain processed grain products (mean concentration, 89.1 ng/g), followed by whole grain rice flour (37.6 ng/g) and whole grain wheat and its products (21.0 ng/g). By contrast, the 4-MBP levels were the highest in whole grain wheat and its products (4.21 ng/g), followed by whole grain processed grain products (2.59 ng/g) and whole grain rice flour (2.13 ng/g). In addition, the BP levels in the F&V juice samples were the highest in 100% reconstituted F&V juices (16.1 ng/mL), followed by F&V juices with a concentration of 10% or more (12.7 ng/mL), and 100% fresh F&V juices (12.4 ng/mL). The BP levels in full-fat milk (8.07 ng/mL) and low-fat milk (8.18 ng/mL) were similar. The levels of 4-MBP in F&V juices were the highest in F&V juices with a concentration of 10% or more (0.70 ng/mL), followed by 100% fresh F&V juices (0.30 ng/mL) and 100% reconstituted F&V juices (0.20 ng/mL). The 4-MBP level in low-fat milk (85.43 ng/mL) was higher than that in full-fat milk (27.56 ng/mL). The levels of M2BB were similar in 100% fresh F&V juices (0.74 ng/mL), 100% reconstituted F&V juices (0.84 ng/mL), and F&V juices with a concentration of 10% or more (0.81 ng/mL). Moreover, the M2BB and PBZ levels in full-fat milk were similar to those in low-fat milk.

In a detailed survey performed by the United Kingdom Food Standards Agency [33], BP (150 ng/g) was detected in 4 of 115 samples of foods packaged in printed plastic (maximum concentration, 0.15 mg/kg), 60 of 296 samples of foods packaged directly or indirectly in printed paper or carton that contained 0.05–3.3 mg of BP/dm^2^ at a concentration of 0.035–4.5 mg/kg (mean concentration, 0.9 mg/kg), and 1 of 54 samples of foods to which a printed sticky label had been attached (at 0.029 mg/kg). In this survey, a high percentage of products in the “frozen foods” (18/35), “jelly” (3/5), and “savory snacks” (15/40) categories tested positive for BP, whereas a low percentage of products in the categories of “sweets, chocolate biscuits, and crisps” (5/35), “bakery products” (8/35), and “cereals” (4/25) tested positive for BP.

According to the United Kingdom Food Standards Agency [35], the potential dietary exposure to BP in high consumption levels consumers is 1.2–1.5 μg/kg bw for adults, which was estimated using the combination of a high level of consumption of foods that may contain BP (449 g/day) with two average levels of consumption of foods containing BP (160 and 200 μg/kg), depending on the different assumptions of values below the LOQ (45 μg/kg) for an adult weighing 60 kg. Samples of skimmed milk, whole milk, and partially skimmed milk packaged in cartons available on the market in the People’s Republic of China were tested. BP was detected in the packaging of all products at a concentration of 0.94–1.37 μg/dm^2^ and in five out of six milk products. Higher levels were detected in milk with a higher fat content, with the levels ranging from 2.84 to 18.35 μg/kg [36]. The migration of BP into five selected dry foods (cake, bread, cereals, rice, and pasta) from a supermarket in Spain was evaluated, and the results revealed that products in the cake category had the highest BP concentration (12 mg/kg) [37].

The migration levels were positively correlated with both porosity and fat content. Our results are in agreement with those reported by Anderson and Castle [38], who found the highest BP concentration (7.3 mg/kg) in high-fat chocolate packaged in printed carton board.

For many chemicals (e.g., plasticizers), the amount that migrates is higher in fatty foods than in low-fat foods. This is attributable to the higher solubility of organic migrants in fat [39,40]. Sanches-Silva et al. (2008) collected 36 samples of commercial beverages (F&V juices, wine, and soft drinks) in Italy, Portugal, and Spain in 2005 and 2006; all the samples were packaged in multimaterial multilayer boxes or aluminum cans [41]. BP was detected in the packaging of four samples (the LOQ in one sample was 1.7 μg/dm^2^ and that in the other three samples ranged from 3.6 to 12.3 μg/dm^2^). On analysis of the samples of beverages contained therein, none yielded positive results for BP detection.

Although fruit juices contain low amounts of fat, photoinitiators can migrate and be adsorbed by the fiber in the juice (fiber content: 0.2%) and thus contaminate the beverage. Many fiber impurities were found in the three F&V juice samples in the present study; nonpolar BP can easily combine with fiber impurities. In previous studies, the highest detection rates and BP levels (2.5–6.5 μg/kg) were observed in fruit juice samples, such as grape, pineapple, and orange juice samples. BP is a component of the film in plastic packaging materials and may be the main photoinitiator unit [42]. The relatively high BP concentration in these three samples could be explained by the presence of fiber impurities in F&V juices and the packaging material.

Recycled cardboard is commonly used for packaging and is in direct contact with dry foods such as flour, pasta, and fast-food items (i.e., foods with short contact durations such as pizzas). Generally, a functional barrier (e.g., plastic or aluminum foil) is used between fatty or aqueous foods and the recycled material to prevent direct contact. Koivikko et al. (2010) reported that the most abundant photoinitiator in nonrecycled products was BP, detected in 61% of samples. In addition, traces of the compound were found in 42% of the samples of the recycled unprinted cardboard. The BP content in these samples varied from 0.57 to 3.99 mg/m^2^. Moreover, the migration level ranged from 1.0 to 18.9 ng/mL in 8 of the 21 samples of recycled paperboard collected [43].

Plastic bags that are used as a barrier against moisture are not always effective [20]. BP can easily migrate through polypropylene films, whereas aluminum and multilayer materials efficiently inhibit BP migration [4,5]. Under low-temperature conditions (−20 °C), BP migrates from cartons to food products during frozen storage, even in the absence of direct contact between the packaging and food or when the packaging is coated with polyethylene [44]. Moreover, because the most commonly used raw material for paperboard is recycled material, the product often contains photoinitiators, including BP. These results indicate that packaging material can affect BP concentration in foods. BP is speculated to be the main photoinitiator and is often used in the production of plastic PE materials and cartons.

Scientists have observed a difference of six orders of magnitude in diffusion coefficient between the worst barrier to diffusion, low-density polyethylene (LDPE), and the best one, polyethylene terephthalate (PET). The poor barrier to benzophenone migration achieved by polyethylene has been experimentally concluded [44,45]. Unlike plastic films, there can be an interaction of the migrants with the carton board because the surface of paper fibres has an overall negative charge due to the carboxy groups from the glucose residues of cellulose and the hydroxy groups of the lignins [46]. Therefore, the electronic affinity for the substrate can play a role in the migration in carton board. Similar to our findings, the chance of potential migration of the photoinitiators introduced in UV–cured ink can be eliminated or, at least, reduced, avoiding the direct contact between the reverse of the printed surface and the food by means of a barrier whose effectiveness will depend basically on their chemical nature and thickness.

Therefore, the BP concentration in Tetra Pak and glass bottles was relatively low in the present study.

### 4.2. Dietary Risk Characterization

Among the six BP-type photoinitiators, the exposure level for BP was the highest in all age groups, followed by that for 4-MBP, whereas the exposure level for M2BB was the lowest in scenario 2.

The estimated dietary exposure levels of BP were higher in the age group of zero to three years than in the other age groups. The median exposure varied from 2.58E-05 mg/kg bw/day (0–3 years) to 4.54E-06 mg/kg bw/day (16–18 years), and the exposure levels in the 97.5 percentile ranged from 2.56E-04 mg/kg bw/day (0–3 years) to 4.11E-05 mg/kg bw/day (16–18 years). In brief, packaged cereals are a major dietary source of exposure to BP among people aged 0–3, 19–65, and >65 years; milk contributes similarly to exposure to PBZ, 4-MBP, 4-OHBP, and 2-OHBP (Figure S2).

The dietary exposure levels were estimated on the basis of a highly conservative scenario in which the maximum concentration observed was assumed to be present in the diet. A comparison between the worst-case dietary exposure estimates of the BP moieties with the highest detected levels in packaged foods and the relevant HBGVs indicates that the food safety risk posed by packaged foods is negligible.

Nondetectable results were assigned a value of zero. The estimated dietary exposure levels to BP in each age group are presented in Figure 2. Dietary risks for the six printing inks or photoinitiators detected using the MOE and TTC approaches are presented in Figure 3, Figure 4 and Figure 5. However, the maximum concentrations of the detected compounds that were considered to be sufficiently conservative were retained in the assessment to ensure that outcomes remained protective of human health. Moreover, although BP is considered a carcinogenic hazard, its lifetime risk is likely to be below 10^−6^ (or one in a million), indicating that it does not pose a substantial dietary risk. The estimated dietary exposure to 2-OHBP was analyzed based on the TTC class III threshold of 1.5 μg/kg bw/day, and the results indicated that it is unlikely to be a safety concern.

The comparison of our estimated dietary exposure levels with those reported in other studies should be interpreted with caution. Large variations may exist in terms of several factors, such as the type of foods included (our study was limited to breakfast foods, including cereals and milk), the analytical method used, the types of food packing materials evaluated, the consumption levels of foods according to the country, and proportion of included individuals with special dietary habits (e.g., vegetarians and vegans).

### 4.3. Comparison of Different Risk Assessment Approaches

In this study, risk assessment was conducted using the RISK21 matrix and the TTC approach. The risk value in the RISK21 matrix was calculated using MOE. For BP and 4-MBP, the BMDL, derived by the EFSA, was considered as the POD to calculate MOE [23]. Compared with the traditional NOAEL approach, BMDL analysis is more reliable because it is less dependent on dose selection and sample size. The BMD approach is currently the US EPA’s preferred dose–response assessment method [47]. Other authorities such as the EFSA have also employed BMD analysis for food safety risk assessment. However, because of the lack of detailed toxicological data on 4-OHBP, PBZ, and M2BB, the traditional NOAEL approach was still applied to calculate the MOE for these compounds in this study. Thus, the BMDL values could not be derived for these compounds.

The TTC approach was used as an early warning assessment tool for the detection of 2-OHBP, which also has no toxicological data. The TTC approach was applied to compare the exposure dose and corresponding threshold values of the compound. In risk assessment, the TTC approach is at a lower tier than other risk assessment approaches such as MOE analysis. To achieve high-tier risk assessment for 2-OHBP, future studies should conduct toxicological tests to obtain complete toxicological data for 2-OHBP.

### 4.4. Strengths and Limitations

The strength of the study was the use of Monte Carlo simulation for probabilistic risk assessment and the RISK21 matrix, which enabled transparent risk assessment, and thereby, effective communication of the risks to scientists, relevant industries, governments, and the public. Moreover, the study used the TTC approach as an early warning assessment tool for the detection of compounds that lack toxicological data.

Two limitations of the study must be considered. First, complete toxicological data was unavailable for some BP-type photoinitiators in this study. Therefore, we could not conduct a high-tier risk assessment for these compounds. Second, the sample size of foodstuffs was small because of insufficient funding for the study.

## 5. Conclusions

In this study, the ADDs of BP-type photoinitiators through food consumption for Taiwanese people were estimated based on two scenarios. The RISK21 matrix and calculated MOE were applied in the carcinogenic and noncarcinogenic risk assessments under two scenarios. In scenario 1 (wherein the SML from the Swiss Ordinance SR 817.023.21 was used), the RISK21 matrix revealed that all MOE values for each BP-type photoinitiator were above the safety limit, indicating acceptable levels for noncarcinogenic and carcinogenic health risks to humans. The cumulative risk in scenario 1 was much higher than the MOE_T_ safety value, indicating an acceptable risk. In scenario 2 (wherein the detected concentration of BP-type photoinitiators was used), the RISK21 matrix indicated that all MOE values were higher than safety limits, indicating acceptable levels for both carcinogenic and noncarcinogenic risks. Because of the lack of complete toxicological data for 2-OHBP, we used the TTC approach as an early warning tool for risk assessment. The estimated ADDs in all age groups were all below the TTC value of 0.0015 mg/kg bw/day (Cramer class III), indicating that 2-OHBP might not exert any adverse effect on human health. Moreover, in scenario 2, the cumulative risk was much higher than the MOE_T_ safety value, indicating an acceptable risk.

The study results revealed acceptable risk levels of exposure to BP-type photoinitiators in the Taiwan population through food consumption of breakfast cereal, fresh milk, and packaged F&V juices. Nevertheless, to improve food safety in Taiwan, regulations for these BP-type photoinitiators could be imposed according to their SMLs proposed by Swiss ordinance. A matter of greater concern to Taiwanese people appears to be the increased risk of exposure to BP-type photoinitiators from other foodstuff sources and exposure routes. Therefore, to obtain a comprehensive exposure profile of Taiwanese people to these photoinitiators, other foodstuffs and exposure routes should be considered in future studies.

## Figures and Tables

**Table 1:** Detection rates for printing ink or photoinitiators in a survey of 362 samples representing different food/packaging combinations.

Foodstuff	Analyte	Detected Rate (%)	Min (ng/g)	Max (ng/g)	Mean (ng/g)	SD (ng/g)	GM (ng/g)
Cereal I: whole grains–rice flour (*n* = 59)	BP	100	17	108	38	20	34
	4-MBP	85	<0.01	12	2.1	1.5	1.9
	4-OHBP	5.1	<0.51	33	17	15	12
	PBZ	1.7	<0.07	0.94	0.94	-	0.94
Cereal II: whole grains–wheat and its products (*n* = 61)	BP	100	14	68	21	8.4	20
	4-MBP	100	1.2	66	4.2	8.3	2.8
	PBZ	1.6	<0.05	0.42	0.42	-	0.42
Cereal III: whole grains–processed grain products (*n* = 60)	BP	100	22	1084	89	199	47
	4-MBP	100	0.90	12	2.6	1.8	2.3
	4-OHBP	17	<0.05	9.7	4.9	2.2	4.5
	M2BB	8.3	<0.29	17.3	5.3	6.9	2.8
100% fresh fruit and vegetable juice (*n* = 14)	BP	100	8.0	29	12	5.4	12
	4-MBP	100	0.20	0.50	0.30	0.08	0.30
	M2BB	93	0.44	0.74	0.57	0.09	1.7
	PBZ	79	0.14	2.3	0.48	0.63	1.5
100% reconstituted fruit and vegetable juice (*n* = 63)	BP	100	7.3	51	16	9.2	14
	4-MBP	87	0.11	1.5	0.20	0.20	0.15
	4-OHBP	1.6	0.82	0.82	0.82	--	0.05
	M2BB	100	0.34	0.84	0.48	0.10	0.47
	PBZ	47.6	0.14	1.6	0.38	0.36	1.2
10% or more fruit and vegetable juice (*n* = 59)	BP	100	5.2	37	13	5.5	12
	4-MBP	95	0.12	8.5	0.70	1.4	0.34
	M2BB	100	0.37	0.81	0.54	0.11	0.52
	PBZ	54	0.14	0.95	0.29	0.19	1.2
Full-fat milk (*n* = 37)	BP	100	4.5	16	8.1	2.3	7.8
	4-MBP	30	0.34	101	28	32	0.17
	4-OHBP	5.4	8.2	12	9.9	2.3	0.14
	M2BB	35	0.22	0.46	0.29	0.09	0.14
	PBZ	57	0.41	2.6	1.6	0.70	0.22
Low-fat milk (*n* = 9)	BP	100	4.2	15	8.2	3.6	7.6
	4-MBP	22	21	150	85	91	0.16
	4-OHBP	11	9.1	9.1	9.1	--	0.17
	M2BB	22	0.26	0.29	0.27	0.02	0.13
	PBZ	78	0.60	2.3	1.6	0.65	0.55

BP = benzophenone; 4-MBP = 4-methylbenzophenone; 4-OHBP = 4-hydroxybenzophenone; PBZ = 4-phenylbenzophenone; M2BB = methyl-2-benzoylbenzoate; SD = standare deviation; GM = geometric mean.

## Data Availability

All data and its supplementary information files generated or analyzed during this study are included in this published article.

## Supplementary Materials

The following are available online at https://www.mdpi.com/article/10.3390/foods11020152/s1, Table S1: Summary of toxicological data of the six BPs and their TTC value; Table S2: TTC classification and corresponding values (in μg/kg bw/day); Table S3: Intake rate of food consumption data for different age groups; Table S4: LOD, recovery, and precision for six benzophenones; Table S5: BP levels in cereals with different types of packaging materials (*n* = 180; ng/g); Table S6: Management regulation of BP-type photoinitiators in food contact materials and printing ink in different countries; Table S7: ADD of exposure to BP-type photoinitiators in different age groups (mg/kg bw/day) (Scenario 1); Figure S1: Sensitivity analysis of exposure to BP-type photoinitiators in the age group of 19–65 years; Figure S2: Contribution of each food group to the ADD of six BPs in all age groups (A) 0–3 years, (B) 3–6 years, (C) 6–18 years, (D) 19–65 years, and (E) >65 years.

## Abbreviations

UV: ultraviolet; SML: specific migration limit; BP: benzophenone; 2-OHBP: 2-hydroxybenzophenone; 4-OHBP: 4-hydroxybenzophenone; 4-MBP: 4-methylbenzophenone; M2BB: methyl-2-benzoylbenzoate; PBZ: 4-phenylbenzophenone; TTC: threshold of toxicological concern; NAHSIT: Nutrition and Health Survey in Taiwan; ECHA: European Chemicals Agency; EFSA: European Food Safety Authority; FaPEx: fast pesticide extraction; NOAEL: nonobserved adverse effect level; ADD: average daily dose; TDI: Tolerable daily intake; LOD: limit of detection; GM: geometric mean; RSD: relative standard deviation; LB: lower bound; UB: upper bound; BW: body weight; IR: Ingestion rate; AF: absorption factor; ME: matrix effect; FCMs: food contact materials; F&V: fruit and vegetable; RCF: Relative Centrifugal Force; LDPE: Low-density polyethylene; PET: polyethylene terephthalate.
