# Supplementary material for: Cumulative Dietary Risk Assessment of Benzophenone-Type Photoinitiators from Packaged Foodstuffs
Source: Foods. 2022 Jan 7;11(2):152. doi: 10.3390/foods11020152 (PMC8774600; doi:10.3390/foods11020152)
Supplement: Supplementary file 1 [file foods-11-00152-s001.zip › foods-1488555-supplementary.pdf]

Table S1. Summary of toxicological data of the six BPs and their TTC value.

	Toxicity data					TTC	
	POD (mg/kg bw/day)	UF	Acceptable MOE	Endpoin	Ref.	Cramer class	Target value (mg/kg bw/day)
BP	$BMDL_{10}$ = 3.1	100	100	Kidney pelvis, transitional epithelium, hyperplasia	EFSA, 2009		
	$BMDL_{10}$ = 18.5	100	100	Kidney, renal tubule, adenoma (Non-genotoxicity cancer)	EFSA, 2009		
2-OHBP	NA	NA	NA	NA	NA	III	0.0015
4-OHBP	NOAEL = 100	100	100	Higher liver weights (Relative to body weights)	ECHA, 2019		
4-MBP	$BMDL_{10}$ = 3.1	200	200	Kidney pelvis, transitional epithelium, hyperplasia	EFSA, 2009		
PBZ	NOAEL = 300	100	100	Decrease in the number of implantation sites	ECHA, 2018		
M2BB	NOAEL = 31.25	100	100	Kidney hyperplasia	ECHA, 2015		

POD: Point of Departure; NA: not available

Table S2. TTC classification and corresponding values (in μg/kg bw/day)

Chemical classification	No. of chemicals	Calculated TTC (μg/ kg body weight/day)	Reference
Genotoxic substances (except aflatoxin-like, azoxy- or N-nitroso compounds)	730	0.0025	Kroes et al.,2004
Organophosphates and carbamates	19	0.3	Kroes et al., 2004
Cramer class I	137	30	Munro et al.,1996
Cramer class II	28	9	Munro et al.,1996
Cramer class III	448	1.5	Munro et al.,1996

Reference

Munro IC, Ford RA, Kennepohl E, et al. Thresholds of toxicological concern based on structure-activity relationships. Drug Metab Rev, 1996, 28(1-2): 209-217.

Kroes R, Renwick AG, Cheeseman M, et al. Structure-based thresholds of toxicological concern (TTC): guidance for application to substances present at low levels in the diet. Food Chem Toxicol, 2004, 42(1):65-83.

Table S3. Intake rate of food consumption data for different age groups.

Food category	Intake rate for different age groups (g/day) Mean±SD (Range)						
	**0-3**	**3-6**	**6-12**	**12-16**	**16-18**	**19-65**	**>65**
**Breakfast cereal**							
Cereal I	2.84±28.33 (0-355.20)	0.45±6.68 (0-100.00)	5.36±3.28 (0-70.29)	0	0.24±2.68 (0-29.77)	0.07±1.47 (0-43.48)	0.29±3.08 (0-41.00)
Cereal II	3.64±20.50 (0-158.43)	1.65±14.40 (0-191.76)	5.36±42.00 (0-512.35)	2.77±29.94 (0-450.96)	0.87±5.97 (0-585.13)	16.22±75.14 (0-861.53)	49.78±127.37 (0-837.88)
Cereal III	1.31±9.43 (0-106.45)	2.43±13.06 (0-142.79)	2.15±14.77 (0-211.38)	3.13±30.24 (0-385.38)	0.73±5.97 (0-59.91)	1.31±23.54 (0-545.71)	2.77±22.00 (0-283.44)
**Fresh milk**							
Full-fat milk	21.73±102.85 (0-1136.42)	42.46±118.08 (0-969.52)	48.48±115.49 (0-888.82)	34.37±105.52 (0-705.52)	29.85±103.63 (0-585.13)	24.02±92.34 (0-976.96)	13.93±71.70 (0-668.30)
Low-fat milk	5.23±57.97 (0-740.14)	10.57±100.38 (0-1398.07)	8.44±43.33 (0-477.08)	10.65±49.75 (0-378.01)	13.03±56.34 (0-435.06)	7.88±46.57 (0-539.89)	2.30±21.39 (0-314.74)
**Packaged F&V juice**							
100% fresh F&V juice	1.16±14.86 (0-190.93)	4.11±43.55 (0-497.79)	4.47±46.38 (0-665.03)	7.59±64.82 (0-665.03)	1.22±10.99 (0-117.70)	6.84±65.58 (0-1083.70)	8.44±114.86 (0-2140.00)
100% reconstituted F&V juice	13.05±69.18 (0-543.73)	20.28±94.82 (0-771.80)	28.54±103.27 (0-665.03)	19.20±126.87 (0-1721.87)	11.55±62.34 (0-433.46)	23.45±125.23 (0-2340.55)	10.80±74.11 (0.-1135.04)
10% or more F&V juice	0.98±12.64 (0-162.35)	0.72±10.85 (0-162.35)	0.06±1.01 (0-20.78)	0	0	1.44±23.51 (0-564.71)	0.40±4.31 (0-56.98)

Data from National Food Consumption Database of NAHSIT in 2019.Cereal I, whole grains-rice flour. Cereal II, whole grains-wheat and its products. Cereal III. whole grains-processed grain products. F&V, fruit and vegetable

Table S4. LOD, recovery, and precision for six benzophenones

Foodstuff	Analyte	LOD (ng/mL)	Recovery (%)	Precision RSD (%)	ME (%)
Cereal I: Whole grains–rice flour (n = 59)	BP	0.001	100	12.4	96
	4-MBP	0.009	69	3.31	112
	2-OH-BP	0.317	113	5.17	64
	4-OH-BP	0.512	90	7.82	96
	M2BB	0.391	29	3.86	97
	PBZ	0.074	35	5.68	81
Cereal II: Whole grains–wheat and its products (n = 61)	BP	0.001	95	11.2	96
	4-MBP	0.009	99	3.39	89
	2-OH-BP	0.142	98	8.61	87
	4-OH-BP	0.043	98	3.47	97
	M2BB	0.277	70	2.78	106
	PBZ	0.046	85	8.37	24
Cereal III: Whole grains–processed grain products (n = 60)	BP	0.001	116	6.77	100
	4-MBP	0.009	102	4.00	108
	2-OH-BP	0.145	84	16.5	100
	4-OH-BP	0.051	110	5.27	87
	M2BB	0.289	57	7.96	89
	PBZ	0.049	49	7.61	84
Fruit and vegetable juice (n = 136)	BP	0.266	105.5	11.3	110
	4-MBP	0.033	103.6	9.40	93
	2-OH-BP	2.176	94.9	10.3	82
	4-OH-BP	0.112	102.4	6.60	105
	M2BB	0.075	106.0	10.9	101
	PBZ	0.040	101.3	8.30	92
Milk (n = 46)	BP	0.502	98.5	3.60	100
	4-MBP	0.093	102.2	5.20	101
	2-OH-BP	1.827	104.9	4.00	101
	4-OH-BP	2.143	110.2	11.8	99
	M2BB	0.065	108.8	10.6	103
	PBZ	0.082	95.9	11.4	101

Abbreviations: ME: matrix effect; RSD: relative standard deviation; LOD: limit of detection

Table S5. BP levels in cereals with different types of packaging materials (n = 180; ng/g)

**Material**	***n***		**Compounds**					
			**4-OHBP**	**M2BB**	**BP**	**2-OHBP**	**4-MBP**	**PBZ**
Iron–aluminum cans	17	DR (%)	0	0	100	0	88	0
		GM	<0.043	<0.277	24.7	<0.142	2.99	<0.046
		AM	<0.043	<0.277	26.9	<0.142	3.83	<0.046
Aluminum foil	108	DR (%)	8	0	100	0	94	1
		GM	6.43	<0.277	27.1	<0.142	2.08	0.42
		AM	9.20	<0.277	29.6	<0.142	2.33	0.42
Plastic	55	DR (%)	7	9	100	0	98	2
		GM	4.14	2.80	46.0	<0.142	2.69	0.94
		AM	4.28	5.34	95.0	<0.142	4.13	0.94

Abbreviations: BP: benzophenone; 2-OHBP: 2-hydroxybenzophenone; 4-OHBP: 4-hydroxybenzophenone; 4-MBP: 4-methylbenzophenone; M2BB: methyl-2-benzoylbenzoate; PBZ: 4-phenylbenzophenone;GM: geometric mean; AM: arithmetic mean; DR: detection rate

Table S6. Management regulation of BP-type photoinitiators in food contact materials and printing ink in different countries.

Country	Regulation, guidelines, standards, and related documents	BP-type photoinitiators	SML (mg/kg)	Remarks
Europe<br>(European Union)	(EC) No1935/2004<br>(EC) No2023/2006<br>(EU) No 10/2011	*Plastic and ink*<br>BP	0.6	
Europe<br>(European Printing Ink Association, EuPIA)	EuPIA Guideline<br>EuPIA GMP<br>EuPIA Suitability List of Photo-initiators for Low Migration UV Printing Inks and Varnishes (2013)	*Printing ink*<br>BP<br>4-MBP<br>M2BB<br>PBZ	 0.6<br>0.6<br>0.05<br>0.01	
Europe<br>(Council of Europe)	Resolution ResAP (2005) 2<br>Technical Document No.1<br>Technical Document No.2 part 1<br>Technical Document No.2 part 2<br>Technical Document No.3	*Printing ink*<br>X		
Germany<br>(Bundesministerium für Ernährung und Landwirtschaft, BMEL)	Twenty-First Ordinance amending the Consumer Goods Ordinance（2017)	*Printing ink*<br>4-MBP	0.05	Total SML of BP and 4-MBP is 0.05 mg/kg
Switzerland<br>(The Federal Department of Home Affairs)	SR 817.023.21（2017)	*Printing ink*<br>BP<br>4-MBP<br>M2BB<br>PBZ<br>*Plastic*<br>BP	 0.6<br>0.05<br>0.05<br>0.01<br><br>0.6	Total SML of BP and MBP is 0.06 mg/kg<br><br>Total SML of BP and MBP is 0.06 mg/kgg
America<br>(Food and Drug Administration, FDA)<br>(National Association of Printing Ink Manufacturers, NAPIM)	CFR - Code of Federal Regulations Title 21	*Packaging and ink*<br>BP is being removed from list		
Japan<br>(Ministry of Health, Labor and Welfare, MHLW)<br>(Japan Printing Ink Makers Association, JPIMA)	Food Sanitation Act 1947 No.223<br>Voluntary Regulation Concerning Printing Inks	*Packaging and ink*<br>X<br>(Negative list)		
China<br>(China Food Safety Regulations)	*GB 9685-2016*	*Ink*<br>BP<br>*Rubber*<br>BP	0.6<br><br>0.6	

"X" means no regulation for BP-type photoinitiators

Table S7. ADD of exposure to BP-type photoinitiators in different age groups (mg/kg bw/day) (Scenario 1)

Age (years)		Average daily dose of BP				
		**Mean**	**SD**	**P2.5**	**P50**	**P97.5**
BP						
	0-3	2.19E-03	3.69E-03	1.56E-04	1.09E-03	1.12E-02
	3-6	2.30E-03	3.19E-03	1.93E-04	1.26E-03	1.12E-02
	6-12	1.61E-03	1.97E-03	1.95E-04	9.64E-04	7.15E-03
	12-16	7.91E-04	1.16E-03	6.30E-05	4.27E-04	3.92E-03
	16-18	4.92E-04	6.99E-04	3.58E-05	2.62E-04	2.47E-03
	19-65	8.86E-04	1.59E-03	5.47E-05	4.17E-04	4.74E-03
	>65	1.01E-03	1.72E-03	6.60E-05	4.93E-04	5.23E-03
4MBP/M2BB						
	0-3	1.82E-04	3.06E-04	1.32E-05	9.02E-05	9.41E-04
	3-6	1.92E-04	2.67E-04	1.62E-05	1.05E-04	9.26E-04
	6-12	1.34E-04	1.61E-04	1.65E-05	8.11E-05	5.90E-04
	12-16	6.57E-05	9.51E-05	5.11E-06	3.55E-05	3.23E-04
	16-18	4.08E-05	5.71E-05	2.98E-06	2.19E-05	2.06E-04
	19-65	7.34E-05	1.38E-04	4.55E-06	3.52E-05	3.92E-04
	>65	8.33E-05	1.51E-04	5.50E-06	4.16E-05	4.18E-04
PBZ						
	0-3	3.65E-05	6.06E-05	2.63E-06	1.82E-05	1.91E-04
	3-6	3.84E-05	5.34E-05	3.29E-06	2.10E-05	1.90E-04
	6-12	2.68E-05	3.26E-05	3.22E-06	1.62E-05	1.18E-04
	12-16	1.30E-05	1.83E-05	1.04E-06	7.08E-06	6.33E-05
	16-18	8.16E-06	1.14E-05	5.58E-07	4.30E-06	4.16E-05
	19-65	1.48E-05	2.66E-05	9.18E-07	6.96E-06	8.07E-05
	>65	1.65E-05	2.68E-05	1.10E-06	8.17E-06	8.70E-05

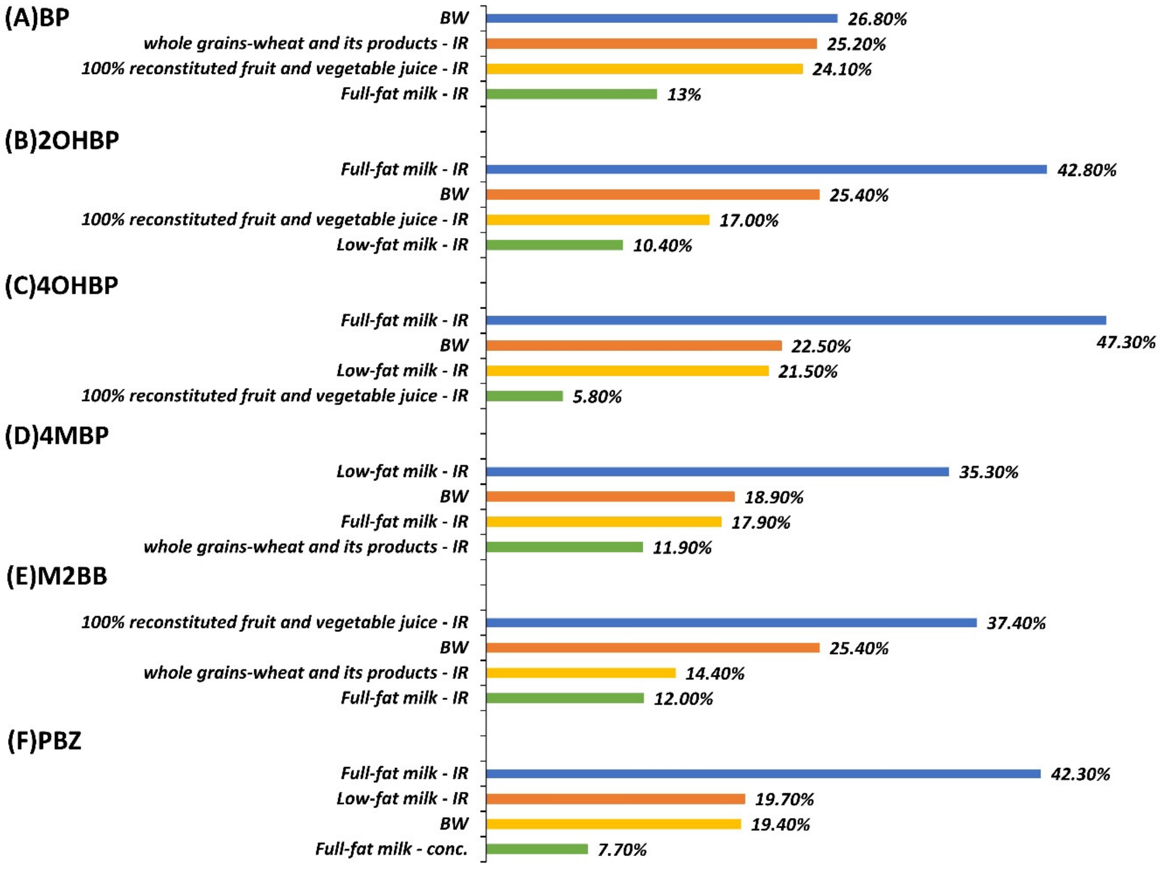

Figure S1. Sensitivity analysis of exposure to BP-type photoinitiators in the age group of 19–65 years.

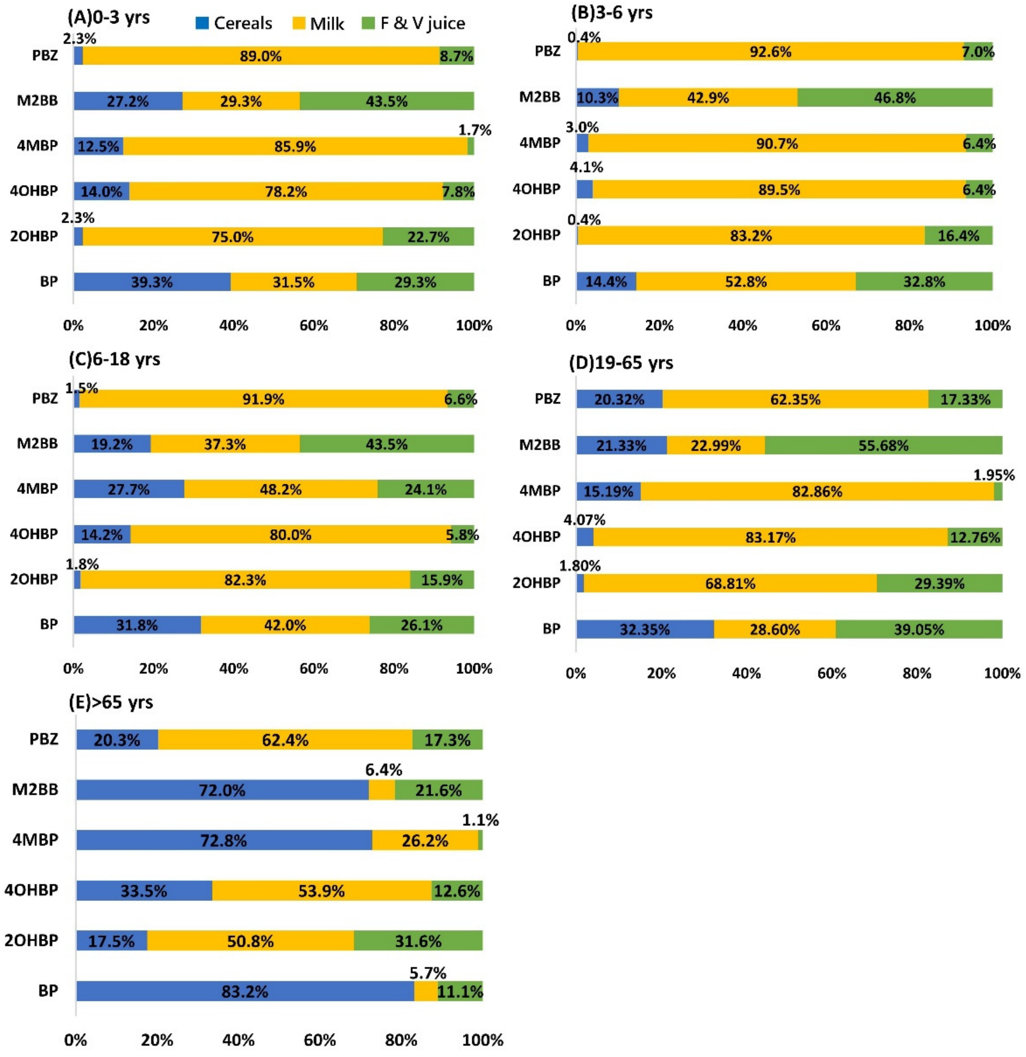

Fig. S2. Contribution of each food group to the ADD of six BPs in all age groups (**A**) 0–3 years, (**B**) 3–6 years, (**C**) 6–18 years, (**D**) 19–65 years, and (**E**) >65 years.

Supplementary Materials-Materials and Methods

Analysis of benzophenone using FaPEx coupled with ultra–high-performance liquid chromatography–tandem mass spectrometry

Fast pesticide extraction (FaPEx) is a method used to extract pesticide residues in agricultural samples by using single-use prefilled sealed cartridges; this method is innovative, simple, and fast and is a simplified version of the QuEChERS method that is based on the same principles (Chuang et al., 2019). We have developed a FaPEx technique coupled with ultra-HPLC (UHPLC)-MS/MS to simultaneously analyze the levels of targeted BPs in the present study. To achieve highly accurate quantification, overcome matrix effects, compensate for method recovery, and mitigate measurement uncertainty, the use of isotope- labeled internal standards (ILISs) for UHPLC-MS/ MS is essential. The developed method was validated and applied to analyze the levels of different BPs in cereal samples according to guidelines established in Taiwan (TFDA, 2013).

All of the linearity, the matrix effect, the limit of detection (LOD), the limit of quantification (LOQ), precision, and accuracy were evaluated and published in our study (Huang et al., 2020). The matrix effect was evaluated through comparison of the slopes of standards in a solvent with matrix-matched standards. LOD and LOQ were defined as the levels with signal-to-noise ratios of 3 and 10, respectively. Blank cereal samples with a 20 ng/g spiking level were used to evaluate the precision and accuracy of the method.

The optimization of UHPLC-MS/MS was also described in our previous study( ). An ESI-positive mode was used for developing multiple analytes, except for BP-2. The retention time, MS parameters (e.g., ion transitions for quantification and confirmation), and collision energy of BPs obtained in the MRM mode. The chromatographic conditions were also optimized. Separation was performed on a Waters Acquity UPLC BEH C18 column, which resulted in a smooth peak and more effective separation of the oatmeal sample with spiking levels of 20 and 8 ng/g for BP standards and ILISs, respectively.

Reference:

Huang, Y.-F.; Chien, J.-T.; Chen, H.-C.; Liu, X.-R.; Chang, J.-P.; Huang, J.-J. Rapid determination of benzophenone derivatives in cereals using FaPEx coupled with ultra-high-performance liquid chromatography-tandem mass spectrometry. *Journal of Food and Drug Analysis* **2021**.

Chuang, W.-C.; Chen, J.-W.; Huang, C.-H.; Shyu, T.-H.; Lin, S.-K. FaPEx® Multipesticide Residues Extraction Kit for Minimizing Sample Preparation Time in Agricultural Produce. *Journal of AOAC International* **2019,** *102*, (6), 1864-1876.

Administration TFDA. Validation Guide of Food Chemical Testing Methods. Taiwan Food and Drug Administration; 2013.
